# Kidney disease and congenital heart disease: Partnership for life

**DOI:** 10.3389/fphys.2022.970389

**Published:** 2022-08-19

**Authors:** Skye El Sayegh, Georges Ephrem, Jay B. Wish, Sharon Moe, Kenneth Lim

**Affiliations:** ^1^ Division of Nephrology & Hypertension, Indiana University School of Medicine, Indianapolis, IN, United States; ^2^ Division of Cardiovascular Medicine, Indiana University School of Medicine, Indianapolis, IN, United States

**Keywords:** cardiorenal, acute kidney injury, chronic kidney disease, congenital heart disease, adult congenital heart disease, cyanotic congenital heart disease

## Abstract

The literature on the relationship between kidney and cardiovascular diseases is continuously expanding. Scientists have elucidated many of the neurohormonal and hemodynamic pathways involved in cardiorenal disease. However, little is known about kidney disease in patients with congenital heart disease. Given advances in the medical and surgical care of this highly complex patient population, survival rates have dramatically improved leading to a higher percentage of adults living with congenital heart disease. Accordingly, a noticeable increase in the prevalence of kidney disease is appreciated in these patients. Some of the main risk factors for developing chronic kidney disease in the adult congenital heart disease population include chronic hypoxia, neurohormonal derangements, intraglomerular hemodynamic changes, prior cardiac surgeries from minimally invasive to open heart surgeries with ischemia, and nephrotoxins. Unfortunately, data regarding the prevalence, pathophysiology, and prognosis of chronic kidney disease in the adult congenital heart disease population remain scarce. This has led to a lack of clear recommendations for evaluating and managing kidney disease in these patients. In this review, we discuss contemporary data on kidney disease in adults with congenital heart disease in addition to some of the gaps in knowledge we face. The article highlights the delicate interaction between disease of the heart and kidneys in these patients, and offers the practitioner tools to more effectively manage this vulnerable population.

## Introduction

Disruptions of the heart-kidney interrelation fall within the realm of an entity called “cardiorenal syndrome”. The medical literature on cardiorenal syndrome is expanding given its clinical implications on morbidity and mortality. (Di Lullo et al., 2017) However, relatively little is known about the cardiorenal interaction in the setting of congenital heart disease (CHD), which imposes a new layer of complexity to this delicate interaction.

CHD affects around 1% of births per year in the United States. (CDC, 2022) CHD can lead to complex physiological changes such as Eisenmenger physiology and Fontan physiology. Eisenmenger physiology encompasses any heart defect with large left-to-right shunting leading to the development of severe pulmonary arterial hypertension and a subsequent right-to-left shunt with resultant significant hypoxemia and cyanosis. Such defects include atrial septal defects, ventricular septal defects, atrioventricular septal defects, and patent ductus arteriosus. Fontan physiology is where patients are born with a single ventricle and receive a palliative heart procedure when a two-ventricle repair cannot be offered. Although the Fontan procedure alleviates cyanosis and ventricular volume overload, patients are left with chronically elevated systemic venous pressure and limited stroke volume augmentation. (d'Udekem et al., 2007) Unfortunately, few data are available regarding the prevalence of CKD in adult congenital heart disease (ACHD) patients as outlined in Table 1. Based on a large study by Dimopoulos et al. conducted at the Royal Brompton Hospital in the United Kingdom (1,102 ACHD patients), the prevalence of CKD was 18- (8.0%) and 35-fold (15.8%) higher in acyanotic and cyanotic CHD (resting oxygen saturations < 90% on room air) respectively compared to the general population. Patients with Eisenmenger physiology, Fontan palliation and patients with more complex anatomy (unrepaired double-outlet and double-inlet ventricle or complex pulmonary atresia) had higher prevalence of CKD than other CHD patients. (Dimopoulos et al., 2008)

**TABLE 1:** Summary of the studies in the literature about CKD in adult patients with CHD.

Study	Number of patients	Patient characteristics	CKD prevalence (eGFR<90 ml/min/1.73 m^2^)	Albuminuria	All-cause mortality risk
Dimopoulos et al., (2008)	1,102	ACHD of various anatomic defects	8% in acyanotic	Not available	5-fold higher than those with normal GFR (Adjusted HR 3.25, 95% CI 1.54 to 6.86, *p* = 0.002)
			15.8% in cyanotic		
Opotowsky et al., (2017)	70	Adults with Fontan procedure	12.9%	23.2 vs. 3.6 mg/g in those with normal GFR	Not available
Wilson et al., (2018)	124	Adults with Fontan procedure	15%	15% had more than 2.5 mg/mmol	Not available
Rajpal et al., (2018)	612	ACHD of various anatomic defects	Not available	17.3% with 30 mg/g or greater	10.4 vs. 1.2% without albuminuria (HR 0.6, 95% CI 0.1 to 5.4, *p* = 0.67)

CKD, chronic kidney disease; eGFR, estimated glomerular filtration rate; ACHD, adult congenital heart disease; HR, hazard ratio; CI, confidence interval.

Rajpal et al. examined the prevalence and prognosis of albuminuria in 612 ACHD patients. (Rajpal et al., 2018) Albuminuria (urine microalbumin-to-creatinine ratio ≥ 30 mg/g) was noted in approximately 1 of 6 patients. Albuminuria among patients with Eisenmenger syndrome or complex cyanotic CHD, simple shunts with clinical sequelae, single-ventricle Fontan, and transposition of the great arteries with a systemic right ventricle, was at least 3-fold more prevalent than in the general population. The higher prevalence of albuminuria was solely related to the underlying CHD, independently of hypertension (HTN) or diabetes mellitus (DM). (Rajpal et al., 2018)

These data highlight the high burden of kidney disease in patients with CHD. Significantly, patients with CHD constitute a unique population requiring combined specialized care by surgeons and cardiologists with expertise in this field. Children born with CHD now undergo increasing cardiac interventions leading to a higher survival rate. This may require a trade-off at the expense of decreased kidney function, whether related to the CHD physiology itself or to the multiple interventions to which the patient may be exposed. Understanding the complexities of cardiorenal interaction in this special population could lead to improvement in their management and outcomes. This review will discuss contemporary data related to CKD in patients with CHD and review the complexities of altered renal physiology in CHD. Furthermore, the article will provide a roadmap for developing prevention strategies, monitoring modalities, and ultimately reversing or delaying progression of kidney disease in this special population by ensuring adequate specialized nephrology care.

## Kidney disease effect on morbidity and mortality

CKD negatively impacts the morbidity and mortality of patients with CHD. Patients with moderate or severe GFR (<60 ml/min/1.73 m^2^) have a 5-fold and 3-fold increase in their 6-year mortality rate compared to those with normal (≥90 ml/min/1.73 m^2^) and mildly reduced GFR (60–89 ml/min/1.73 m^2^) respectively. (Dimopoulos et al., 2008) When examining the effect of albuminuria on mortality, Rajpal et al. noted a stepwise increased risk of death and nonelective hospitalization for cardiovascular causes with increasing albuminuria. This risk remained unchanged after adjusting for age, cyanosis, DM, New York Heart Association functional class level, disease complexity, and eGFR. (Rajpal et al., 2018)

Interestingly, Rajpal et al. noted that albuminuria was associated with adverse outcomes and mortality risk among patients with biventricular circulation, but not those with single-ventricle physiology (Fontan). The authors also examined 404 adults with CHD who underwent cardiopulmonary exercise testing (CPET) and noted that albuminuria was associated with a significantly lower peak oxygen consumption (VO_2_Peak, and index of cardiovascular functional capacity or cardiorespiratory fitness) and less efficient ventilation; this association was present only among patients with biventricular physiology but not with Fontan circulation. (Rajpal et al., 2018)

Unfortunately, the data is biased, as patients with renal dysfunction may be less likely to be offered cardiac surgeries or other invasive interventions that could be indicated due to the increased perioperative risk with the renal impairment.

### Congenital heart disease altering kidney physiology

**FIGURE 1:**
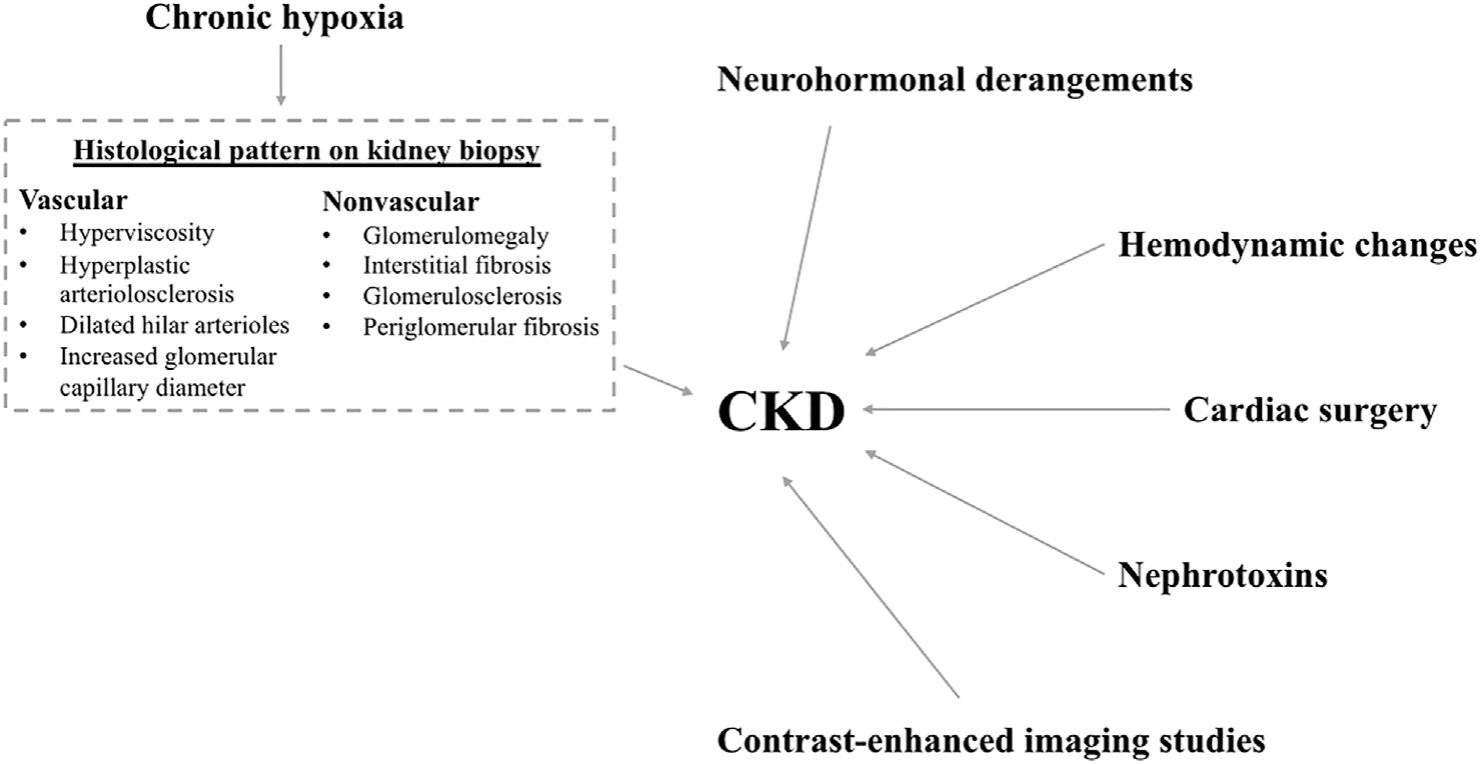
Factors leading to chronic kidney disease. Figure legend: Schematic diagram showing the multiple risk factors for CKD development. These risk factors include: neurohormonal derangements, hemodynamic changes, cardiac surgery, nephrotoxins, contrast-enhanced imaging studies and chronic hypoxia. The histological changes seen on kidney biopsy of patients with chronic hypoxia include vascular and nonvascular changes as listed in the dashed text box.

Children and adults with CHD are exposed to multiple causal pathways that can lead to CKD development as illustrated in Figure 1. These include chronic hypoxia, intraglomerular hemodynamic changes, neurohormonal derangements, cardiac surgery and nephrotoxins. (Morgan et al., 2015)

Glomerular enlargement on light microscopy of kidney tissue in patients with cyanotic CHD with chronic hypoxemia and hyperviscosity was first noted in 1953, leading to the term “cyanotic nephropathy”. (Lee, 2019) The physiological changes that occur in the kidneys of patients with cyanotic CHD are complex and not fully understood. On autopsies of 50 kidneys from children with cyanotic CHD, Gupte et al. found increased glomerulomegaly, glomerulosclerosis, periglomerular fibrosis, hyperplastic arteriolosclerosis, and interstitial fibrosis compared to age-matched controls. (Gupte et al., 2014) Perloff et al. examined the kidneys of 288 patients with cyanotic CHD by light microscopy and electron microscopy. They were able to identify two glomerular histological patterns, vascular and nonvascular, and proposed two pathogenetic mechanisms: In the vascular pattern they found dilated hilar arterioles, significant increase in glomerular capillary diameter with red cell engorgement, and overall glomerular enlargement. These findings suggested that erythrocytosis leads to higher viscosity; high viscosity leads to an increase in endothelial shear stress and glomerular vascular resistance, stimulating intraglomerular release of nitric oxide which in turn results in glomerular vascular bed dilation leading to glomerular hyperfiltration. In the nonvascular glomerular abnormality, they observed an increase in juxtaglomerular and mesangial cellularity and an increase in mesangial matrix. This suggests that megakaryocytes circulating *via* right-to-left arterial shunts are dislodged in glomeruli where they release high concentrations of platelet-derived growth factor (PDGF) and transforming growth factor-β (TGF-β) which are likely responsible for the nonvascular abnormality above. (Perloff et al., 2000)

Albuminuria or proteinuria are frequently observed once glomerular enlargement occurs, which is known to be associated with progressive decline of kidney function and glomerulosclerosis. (Lee, 2019) Fang et al. demonstrated that 46.5% of 359 children with CHD developed CKD, defined as estimated glomerular filtration rate (eGFR) < 90 ml/min/1.73 m^2^ on two consecutive tests. This study also found that patients with cyanotic CHD compared to acyanotic CHD had almost double the rate (61 vs. 37%) of clinically relevant CKD, defined as eGFR <60 ml/min/1.73 m^2^, urine protein-to-creatinine ratio (UPCR) > 0.5 g/g, or urine albumin-to-creatinine-ratio (UACR) > 30 mg/g. The authors also noted a significantly higher incidence of CKD among patients who received palliative compared to corrective heart surgery (44 vs. 13%), and those who received more contrast-enhanced imaging studies (61 vs. 31%). (Fang et al., 2021) To better understand the association of CKD incidence and complexity of cardiac surgery, Nakae et al. examined CKD incidence after 2 years of open-heart surgery for CHD in 147 patients under the age of 5. The authors classified surgical complexity into six categories (1 through 6) of increasing mortality risk using the Risk Adjustment for Congenital Heart Surgery-1 (RACHS-1) classification. They noted that compared to patients without CKD, patients with CKD had higher RACHS-1 category (2 vs 3) and that high RACHS-1 category was an independent risk factor for developing CKD (OR 1.7; 95% CI 1.0–2.9; *p* = 0.041). (Nakae et al., 2022)

In a study by Wilson et al., the investigators examined kidney function in 124 patients with Fontan circulation using *in vivo* Tc-99m DTPA measurement of GFR and UACR. Fifty-seven percent of subjects had either measured GFR <90 ml/min/1.73 m^2^ or an elevated UACR. (Wilson et al., 2018) Awad et al. studied the physiological changes that contribute to nephropathy in 86 children with cyanotic CHD. (Awad et al., 2003) Increased duration of cyanosis was associated with a significant elevation of urinary markers of glomerular and tubular dysfunction. Glomerular function was studied by measuring urinary total protein and microalbumin; tubular function was assessed by urinary alpha-1-microglobulin; structural integrity of the renal proximal tubules was studied by measuring urinary activities of the brush-border enzyme leucine-aminopeptidase and the lysosomal enzyme N-acetyl-beta-D-glucosaminidase. Furthermore, patients who underwent palliative surgery exhibited a significant decrease in these urinary biomarkers suggestive of improved nephropathy. Palliative surgery in patients with cyanotic CHD significantly improves oxygen saturation and decreases hematocrit level and blood hyperviscosity leading to acute and reversible changes in renal hemodynamics. (Awad et al., 2003) These results suggest that patients with cyanotic heart disease are at a greater risk of developing CKD and palliative or corrective heart surgery at an early age may slow progression of CKD or facilitate renal recovery.

### Surgery, nephrotoxin exposure and kidney complications

Several studies examined the prevalence and risk factors for developing acute kidney injury (AKI) after CHD surgery in children. They have also examined the long-term relationship between CHD surgery in children and the risk of developing CKD. The greatest challenge in collectively summarizing the incidence of AKI and CKD is the heterogeneity in defining AKI and CKD. These studies have used Acute Kidney Injury Network (AKIN), Kidney Disease Improving Global Outcomes (KDIGO) or Pediatric Risk, Injury, Failure, Loss, End-Stage Renal Disease (pRIFLE) classifications for their definitions of AKI which has been largely heterogenous. A meta-analysis performed by Van den Eynde et al. included 61 studies examining cardiac surgery associated AKI in pediatric patients (<18 years old) showed that the pooled estimated incidence of AKI was 34.3% (95% confidence interval 30.0–38.8%, I^2^ = 96.8%). (Van den Eynde et al., 2022a) Overall, 30–60% of children with CHD undergoing cardiac surgery develop AKI leading to increased length of stay, morbidity, and mortality. Another meta-analysis presented by Van den Eynde et al. included 58 studies investigating in-hospital outcomes of pediatric patients (<18 years old). This meta-analysis demonstrated that patients with cardiac surgery associated AKI had increased pediatric intensive care unit and hospital length of stay, longer ventilation times, higher need for renal replacement therapy, increased risk of cardiac arrhythmias and higher rates of in-hospital mortality. (Van den Eynde et al., 2021)The most notable risk factors for developing AKI included younger age, lower body weight, prolonged cardiopulmonary bypass time, perioperative fluid overload, and the presence of low cardiac output after surgery. (Khuong et al., 2021) In addition, in the meta-analysis by Van den Eynde et al., the authors found that other risk factors for developing AKI included presence of pulmonary hypertension, cyanotic heart disease, univentricular heart, RACHS-1 score ≥ 3, vasopressor use, cardiopulmonary bypass use, reoperation, and sepsis. (Van den Eynde et al., 2022a)

Fuhrman et al. reported that persistent kidney dysfunction on discharge was noted in one third of 195 ACHD patients admitted to cardiac intensive care units who developed AKI. Moreover, the authors reported that all patients who died within the first year after hospital discharge had CKD. (Fuhrman et al., 2021) Another study by Kwiatkowski et al. reported a 36% incidence of AKI among ACHD patients undergoing cardiac surgery. Significant risk factors for AKI were older age (≥35), underlying left ventricular dysfunction or arrhythmia, longer cardiopulmonary bypass time, and perioperative vancomycin use. AKI was associated with a prolonged duration of mechanical ventilation and intensive care unit stay. (Kwiatkowski et al., 2017)

The Translational Research Investigating Biomarker Endpoints in AKI (TRIBE-AKI) study is one of the largest prospective studies exploring the relationship between AKI and CKD in children undergoing CHD surgery requiring cardiopulmonary bypass. Fifty-seven of 131 children had postoperative AKI. This study demonstrated a high prevalence of CKD (22%), defined as impaired eGFR (<90 ml/min/1.73 m^2^) or microalbuminuria (UACR >30 mg/g), and hypertension (HTN) (17%) at 5 years follow up. Interestingly, this high prevalence of CKD and HTN was not specifically associated with postoperative AKI. Patients who developed CKD sustained more kidney insults including nephrotoxins and more cardiac surgeries and contrast enhanced studies. (Greenberg et al., 2016) A Danish study by Madsen et al. of 382 patients with CHD undergoing cardiac surgery, demonstrated that the 5-year cumulative incidence of CKD was 12% for patients with cardiac surgery-associated AKI compared to 3% in those with non-cardiac surgery-associated AKI. (Madsen et al., 2017) Another large study by Parikh et al. examined the incidence of end stage kidney disease (ESKD) and mortality after pediatric cardiac surgery in 3,600 children with CHD over a median follow up of around 6 years. Four percent of children who had surgery for CHD died and 1% reached ESKD. Risk of death or ESKD or both increased with severity of CHD, the highest in children with hypoplastic left heart syndrome. (Parikh et al., 2019) Van den Eynde et al. performed a prospective cohort study in which they noted that 113 out of the 571 children (<16 years old) who underwent cardiac surgery developed AKI. After approximately 5 years follow up, the authors performed a formal kidney assessment in 66 of the children that developed cardiac surgery related AKI. Of the 66 patients, 39 had at least 1 marker of kidney injury. Biomarkers of kidney injury that were noted included: 13.6% had reduced kidney function (eGFR <90 ml/min per 1.73 m^2^), 0.9% had proteinuria, 7.6% had α1-microglobinuria, 19.7% had hypertension. (Van den Eynde et al., 2022b)

Several studies examined nephrotoxin exposure, which can be considered a modifiable risk factor, and its effects on kidney dysfunction or CKD. A retrospective cohort study by Uber et al. including 154 children reported that 85.1% of them had received at least one nephrotoxin and 20.8% ≥ 3 nephrotoxins. The most frequently administered drugs that have been associated with nephrotoxicity were ketorolac, aspirin, ibuprofen, vancomycin, piperacillin/tazobactam, and enalapril in the setting of volume depletion. Even though AKI was more common in children receiving ≥ 3 nephrotoxins, it was not statistically significant after adjusting for confounders. (Uber et al., 2018) In a retrospective study by Phelps et al., the investigators evaluated the role of furosemide alone or in combination with angiotensin-converting enzyme (ACE) inhibitors and the risk of development of AKI in 319 children undergoing cardiac surgery. The study found that administration of furosemide alone or in combination with an ACE inhibitor was not associated with an increased incidence of AKI. (Phelps et al., 2012)

As described above, patients receiving cardiac interventions, contrast enhanced studies and nephrotoxins are more likely to develop AKI and/or CKD. However, certain cardiac interventions and contrast enhanced studies may be clinically indicated even if that entails an increased risk of kidney complications. This raises the importance of carefully assessing the risk versus benefit ratios of these interventions and encouraging collaboration between cardiologists and nephrologists to develop an ideal plan of care that ultimately benefits the overall health of the patient with the least possible risk or complications.

### Assessment of kidney disease in adult congenital heart disease

To assess and monitor kidney disease in patients with CHD, standard diagnostic studies must be available to adequately identify the presence and severity of kidney disease. This will allow for monitoring the effectiveness of strategies to avoid or delay CKD progression by providing the clinician with reproducible data trends.

Currently used diagnostic biomarkers for kidney disease include the newly implemented eGFR 2021 CKD Epidemiology Collaboration (CKD-EPI) creatinine equation without race, in addition to CKD-EPI cystatin C equation and the measurement of UAC. A large prospective cohort study by Opotowsky et al., in 2019 compared creatinine-based eGFR to cystatin C-based eGFR in ACHD patients from the Boston Adult Congenital Heart Disease Biobank. In this population, cystatin C-based eGFR was a significantly better predictor than creatinine-based eGFR for clinical events including all-cause mortality. The poor agreement between creatinine-based and cystatin C-based eGFR was especially notable in patients with Fontan circulation. (Opotowsky et al., 2019)

Opotowsky et al. examined how eGFR and urinary biomarkers of kidney injury are altered in patients with Fontan circulation aged 18–54 years. Fifty-nine patients were reported with urine biomarker data and 70 patients were reported with eGFR data using serum creatinine and cystatin C. Approximately 13% of Fontan patients had eGFR by cystatin C < 90 ml/min/1.73 m^2^ as compared to 0% of general population. This was more discriminating than eGFR by creatinine where no difference between Fontan patients and the general population was reported. Fontan patients had significantly higher UACR (23.2 vs. 3.6 mg/g), N-acetyl glucosaminidase (NAG) (1.8 vs 1.1 U/g) and KIM-1 concentrations (0.91 vs. 0.33 ng/mg) but no significant difference in urinary neutrophil gelatinase-associated lipocalin (NGAL) concentration compared to normal controls. NAG, KIM-1 and NGAL are few of the novel biomarkers in development. An increased incidence of a composite endpoint for non-elective cardiovascular hospitalization or death was noted among Fontan patients with reduced eGFR by cystatin C, elevated urinary KIM-1 and elevated urinary NAG but not in patients with microalbuminuria. (Opotowsky et al., 2017)

In a pilot case control study conducted at Children’s Hospital of Pittsburgh Adult Congenital Heart Disease Center, the investigators compared baseline concentration of tubular biomarkers between 30 young adults with CHD and 30 healthy young adults with the aim of detecting subclinical kidney injury. Both groups had no clinically significant proteinuria nor albuminuria. The group with CHD had normal kidney function including serum creatinine and eGFR. None of the patients with CHD had documented hypoxemia, which is known to negatively impact renal function. This study showed that, patients with CHD compared to patients without CHD, had significantly higher urinary levels of kidney injury molecule-1/creatinine (KIM-1/Cr) and significantly lower urinary concentrations of tissue inhibitor of metalloproteinases-2 (TIMP-2) and insulin-like growth factor binding protein 7 (IGFBP7). These biomarkers could potentially be used to detect early tubular injury before abnormalities in traditional biomarkers of kidney dysfunction are apparent. (Fuhrman et al., 2019)

The identification of more accurate and sensitive diagnostic tools and biomarkers of kidney function is currently a research priority. Further studies that include younger patients with CHD are needed as currently used biomarkers were primarily validated in adult patients. More reliable identification of kidney dysfunction would allow earlier intervention to delay progression of kidney disease such as targeting renin-angiotensin-aldosterone system and properly dosing medications to minimize nephrotoxin exposure. Other benefits of accurate estimation of kidney function include more precise peri-operative risk stratification, and more personalized care including guiding the appropriate use of radiocontrast studies and routine catheterizations, and timely consideration of heart or heart and kidney transplant evaluations.

### Management of kidney disease in adult patients with congenital heart disease

Based on currently available evidence, we recommend assessing eGFR by cystatin C at first visit in addition to obtaining a routine renal panel and UACR. If eGFR by cystatin C is < 60 ml/min/1.73 m^2^ and/or if albuminuria is present, we recommend earlier referral to nephrology to assist in the care of these complex patients. Frequency of monitoring of renal function should be performed at the discretion of the nephrologist on a case-by-case basis given anatomical variability of heart defects and the type of heart surgeries performed. We recommend special attention to patients with cyanotic CHD given their significantly increased risk of developing CKD compared to their acyanotic counterparts. We also recommend closer follow up of patients requiring subsequent cardiac surgeries and/or prolonged hospitalizations with particular emphasis on checking eGFR by cystatin C rather than eGFR by creatinine within 3 months of these events.

Unfortunately, there are no evidence-based recommendations regarding the management of kidney disease in ACHD patients. This is in part due to the paucity of published data, and the small sample sizes of the available ones. More specifically, there are no published guidelines for the selection and frequency of monitoring kidney disease in ACHD patients. Given the higher risk for CKD and its associated adverse outcomes in this population, it would seem prudent to perform regular screening with measurement of eGFR and UACR at every visit, at least annually for stable patients or biannually for patients with severe CHD or unstable kidney function with referral to a nephrologist for more specialized testing and risk-stratification should CKD be present.

It is reasonable to speculate that the available and well-studied data demonstrating slowing or prevention of kidney disease progression in the general adult population can also be applied to this specific younger population, including the use of renin-angiotensin-aldosterone system blockade agents (RAAS inhibitors), mineralocorticoid receptor antagonists (MRAs), and sodium-glucose co-transporter 2 (SGLT2) inhibitors. We are currently in need of well-designed randomized trials to assess the benefits of these pharmacologic agents in ACHD patients with CKD. Meanwhile, we recommend that nephrologists assist cardiologists in optimizing volume status and hemodynamics with close monitoring of medication dosing per renal function and optimizing medications perioperatively if needed.

## Conclusion and future directions

With rapidly advancing medical therapies, patients with CHD are living longer allowing previously unseen complications to occur in other organs including the kidneys. Understanding the impact on the kidneys of CHD and its treatments, whether medical or surgical, may allow healthcare providers to prevent the development of kidney disease and/or delay its progression in ACHD patients. Further studies on physiological changes that lead to kidney impairment secondary to CHD and resulting ramifications are severely lacking. Moreover, there are no evidence-based recommendations on how to best manage CKD in ACHD patients.

Despite these limitations, recommendations can be made: First, the assessment of kidney function using eGFR by cystatin C testing in this population is warranted due to alterations in physical activity and growth affecting muscle mass leading to altered creatinine unrelated to true GFR. Second, at least annual assessment of UAC is indicated as an early biomarker of CKD while we await research into other novel biomarkers. Third, we need collaboration between nephrology and cardiology to optimize hemodynamics and volume status of these patients, tailor medications appropriately to eGFR and help address risks of contrast and other nephrotoxins. Such recommendations may also encompass a more conservative approach to the use of radiocontrast imaging, cardiac catheterizations, consecutive surgeries, and potentially nephrotoxic medications in patients based on risk-stratification and the degree of kidney disease present.

As nephrologists and cardiologists, we should work on further strengthening our partnership to better understand and manage the cardiorenal syndrome in ACHD patients with CKD, which differs from other forms of cardiorenal syndromes. This can be achieved by including CHD on the curriculum of nephrology fellowship training as it is largely absent at present, integrating and expanding the awareness of kidney disease and care in the curriculum of CHD training among cardiology fellows, and by convening multidisciplinary meetings involving nephrologists, cardiologists, pharmacists, and social workers at the local and national level.

### Key learning points

• Patients with CHD are living longer, allowing previously unseen complications to occur in other organs including the kidneys.
• Patients with cyanotic CHD are at a significantly increased risk of developing CKD compared to patients with acyanotic CHD.

### Proposed recommendations

• Assess eGFR by cystatin C in addition to obtaining a routine renal panel and UACR at first visit. We recommend repeating these tests annually for stable patients or biannually for patients with severe ACHD or unstable kidney function.
• If eGFR by cystatin C is < 60 ml/min/1.73 m^2^ and/or if albuminuria is present, we recommend earlier referral to nephrology for more specialized testing and risk-stratification.
• Frequency of monitoring of renal function remains at the discretion of the nephrologist on a case-by-case basis, given anatomical variability of heart defects and the type of heart surgeries performed.
• Patients requiring subsequent cardiac surgeries and/or prolonged hospitalizations necessitate closer follow up with checking eGFR by cystatin C rather than eGFR by creatinine within 3 months of these events.
• Slowing or preventing kidney disease progression by considering the use of RAAS inhibitors, MRAs, and SGLT2 inhibitors.
• We encourage collaboration between nephrology and cardiology to optimize hemodynamics and volume status of these patients, tailor medications appropriately to eGFR and help address risks of contrast and other nephrotoxins.
